# Quo Vadis, Amadeo Hand Robot? A Randomized Study with a Hand Recovery Predictive Model in Subacute Stroke

**DOI:** 10.3390/ijerph20010690

**Published:** 2022-12-30

**Authors:** Pedro Amalio Serrano-López Terradas, Teresa Criado Ferrer, Iris Jakob, Jose Ignacio Calvo-Arenillas

**Affiliations:** 1 Robotics Unit, Brain Damage Service, Hospital Beata María Ana, 28007 Madrid, Spain; 2 Centro Superior de Estudios Universitarios La Salle, Universidad Autónoma de Madrid, 28023 Madrid, Spain; 3 Occupational Thinks Research Group, Occupational Therapy Department, Centro Superior de Estudios Universitarios La Salle, Universidad Autónoma de Madrid, 28049 Madrid, Spain; 4 Tyromotion, GmbH, 8020 Graz, Austria; 5 Facultad de Enfermería y Fisioterapia, Universidad de Salamanca, 37008 Salamanca, Spain

**Keywords:** Amadeo^®^, hand, occupational therapy, rehabilitation, robotics, stroke

## Abstract

Background. Early identification of hand-prognosis-factors at patient’s admission could help to select optimal synergistic rehabilitation programs based on conventional (COHT) or robot-assisted (RAT) therapies. Methods. In this bi-phase cross-over prospective study, 58 stroke patients were enrolled in two randomized groups. Both groups received same treatments A + B (A = 36 COHT sessions for 10 weeks; B = 36 RAT sessions for 10 weeks; 45 min/session; 3 to 5 times per week). Outcome repeated measures by blinded assessors included FMUL, BBT, NHPT, Amadeo Robot (AHR) and AMPS. Statistical comparisons by Pearson’s rank correlations and one-way analyses of variance (ANOVA) with Bonferroni posthoc tests, with size effects and statistic power, were reported. Multiple backward linear regression models were used to predict the variability of sensorimotor and functional outcomes.Results. Isolated COHT or RAT treatments improved hand function at 3 months. While “higher hand paresis at admission” affected to sensorimotor and functional outcomes, “laterality of injury” did not seem to affect the recovery of the hand. Kinetic-kinematic parameters of robot allowed creating a predictive model of hand recovery at 3 and 6 months from 1st session. Conclusions. Hand impairment is an important factor in define sensorimotor and functional outcomes, but not lesion laterality, to predict hand recovery.

## 1. Introduction

Improving hand hemiparesis of the upper limb (UL) and its functional impact after stroke remains the main objective in neurorehabilitation. At four years after the stroke, only 4% of patients are satisfied with the functionality achieved, considering chronic deficit in almost 50% of cases [1]. Location, extension and severity of the neurological lesion, more frequent in the middle or anterior cerebral arteries, entail heterogeneity of sensorimotor, cognitive, emotional and functional signs and symptoms that condition different types of neurological hand syndromes, determining the degree of disability of the person and their subsequent level of autonomy achieved in everyday life. Atypical clinical pictures of anosognosia, somatoparaphrenia or alien hand can occur after acquired brain damage (ABI), in addition to the more typical hand syndromes of apraxia or neglect.In the early stages of recovery, a better prognostic factor is an initial capacity for distal activation of the hand in one or more fingers and a lower presence of sensitivity impairment [1,2].The presence of a greater degree of motor and somatosensory involvement in the UL at the beginning of the intervention, as well as the location and lateralization of the injury, could determine the level of recovery of hand functionality from the subacute phase [3].

Intervention from this phase in these heterogeneous neurological hand syndromes, currently still with an uncertain prognosis and slow recovery, should be aimed at reducing not only the deficiencies in the cognitive and sensorimotor components of the UL, but also the impact that the deficits due to hemiparesis cause in the performance of activities of daily living (ADLs). Early evaluation and adequate multidisciplinary intervention can contribute to accelerating functional recovery and improving the quality of life of these patients, reducing co-morbidity and the risk of death [4,5]. This includes emerging assessment and treatment tools, novel techniques in the areas of neuroimaging and structural, chemical and functional neuropsychology, advances in the field of cell-based and genetic therapies, and promising new techniques for motor and cognitive restoration, as well as the use of electronic technologies as adjuncts to conventional occupational therapy and physical therapy, including assistive devices, virtual reality (VR), and robotics (RAT) [6,7,8].

These technologic devices, virtual or robotics in continuous development, progressively integrate new concepts based on learning and motor control theories into their design. They intend to stimulate the most complex voluntary motor control centers and mirror neuron network, by increasing high-frequency afferent somatosensory stimulation which, added to the increase in efferent activity of the affected hemibody, could contribute to activating neuroplasticity processes, stimulating the reorganization of the functional brain map, improve the interhemispheric disequilibrium produced by the injury and facilitate the processes of diaschisis or synaptogenesis, both in the ipsilateral and contralateral sensorimotor cortex [1,9]. Unfortunately for some stroke patients, the presence of severe sensorimotor deficits on admission could lead to their exclusion from this type of intervention. Most studies do not provide indicators of the effect of transfer or generalization of learning to the functional context, although they show evidence of sensorimotor improvement [10,11].

Some evidence shows that improvements at the proximal level do not migrate to the distal level or vice versa, making a deficit-specific approach to the paretic arm more necessary to optimize improvements in specific proximal or distal motor skills, through a practice based on intensive, repetitive, motivating and high-frequency exercises, as new technologies could provide compared to the conventional approach [12,13].

Additionally, there is growing evidence on the use of robotics in stroke and other neurological diseases, as an adjunct to the more traditional rehabilitation of the UL, both in children and adults [9,10,14,15]. Both proximal and distal approaches to the neurologic hemiparesis, or combined, are possible with these robotic devices [16]. Among all the exoskeleton and end-effector robotic devices, Amadeo^®^ hand robot (AHR) turns out to be the robot with the most available evidence for the approach of the neurological hand in stroke. The literature shows it as a safe, easy-to-use, adjustable, reliable and valid tool for the treatment of the grip movement and the improvement of the kinetic and kinematic parameters related to the flexo-extension of the fingers in the biomechanical end-effector of the upper limb, allowing its use in combination with other technological techniques (EEG, sEMG) or traditional occupational (OT) or physiotherapy (PT) neurorehabilitation approaches, which improves its augmented feedback [15,16].

Currently there are few predictive models for recovery of the hemiparetic upper limb after stroke, and in particular there are even fewer that attempt to predict the behavior that some predictor variables could have on specific criterion variables associated with the domain or function of the hand, usually the core of the intervention from occupational therapy [17,18,19,20,21,22].

In this research, after performing a qualitative and quantitative analysis of the best available evidence in a previous doctoral work [23], we focus on the results of a 5-year randomized study of clinical application with the AHR in the Robotics Unit of the Beata María Ana Hospital´s Brain Injury Service, that test the added value of this device as a primary assessment tool in an upper limb protocol, as well as in the creation of its own novel predictive clinical model (AMADEUS) that complements the IS-BRAIN synergistic interventional theoretical model, in which robotic therapy evolves from an *ad libitum* application to an *ex juvantibus* dosage-model. There are no precedents of studies applied with this robot that investigate its effect in a large sample of patients grouped by paresis or in relation to the presence of negligence or apraxia that affects manual function.

## 2. Materials and Methods

### 2.1. Study Design and Randomization

**Figure 1:**
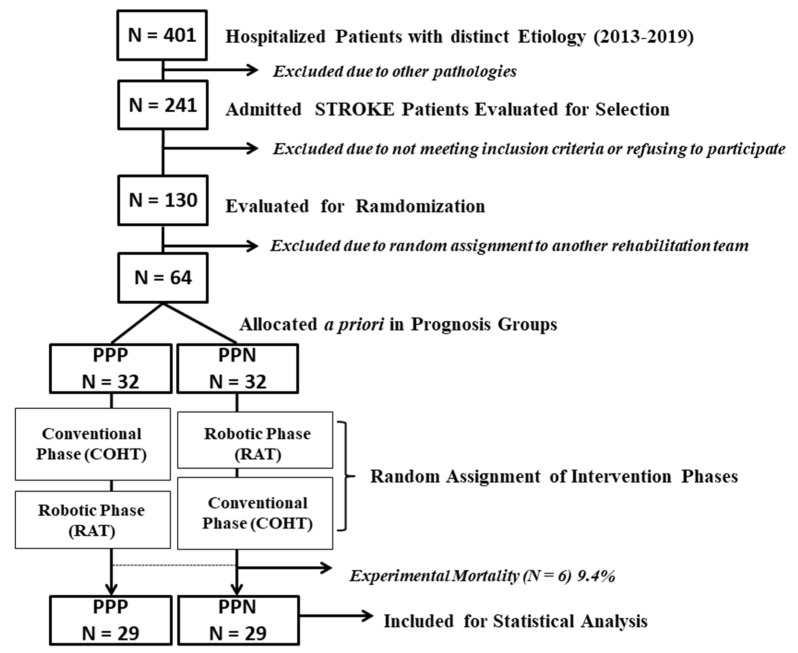
CONSORT flow chart identifying the phases of this study: Selection, recruitment, monitoring, and analysis processes are shown. COHT: conventional hand-specific occupational therapy; PPP: positive prognostic profile group; PPN: negative prognostic profile group; RAT: robot-assisted therapy.

An experimental, longitudinal, prospective, randomized, controlled, biphasic, crossover design study has been implemented. Fifty-eight patients were distributed to receive in each case, and in a randomly assigned sequential order, 2 different treatment modalities AB or BA. Treatment A (control) consisted of 36 sessions for 10 weeks, based on occupational therapy approaches (COHT). Treatment B (experimental) consisted of another 36 sessions over 10 weeks with the AHR version 5 (RAT). Each treatment session, A or B, was 45 min. The same intensity and frequency were established for the control and experimental phases. Each intervention phase was designed ad hoc with a length of 3 months. A washout period of 2 weeks was defined between experimental conditions. Control and experimental treatment groups were matched on research variables. The processes of selection, recruitment, monitoring and analysis of the study sample can be seen in the CONSORT flowchart (see Figure 1).

### 2.2. Participants

**Figure 2:**
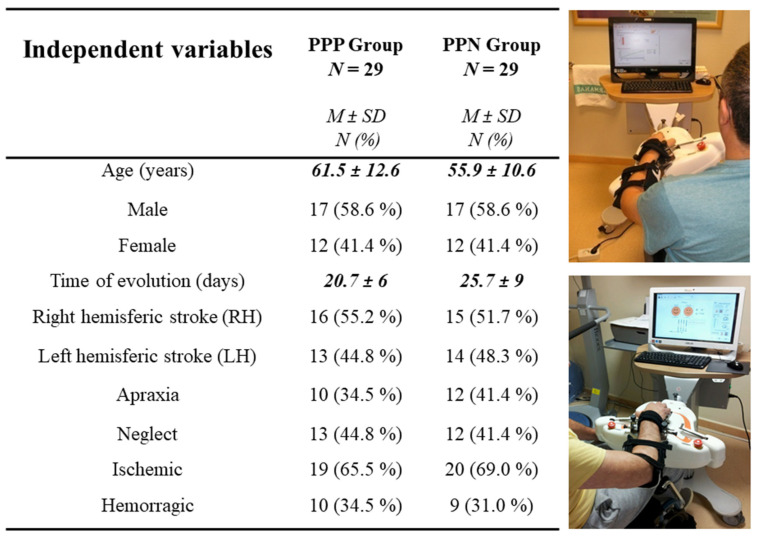
Demographic and clinical characteristics of the experimental groups. PPP: positive prognosis profile; PPN: negative prognosis profile; M:mean; SD: standard deviation; N: sample size.Statistical significant differences were found between variables in black (*p* < 0.000).

A control group of healthy volunteers (*n* = 30) matched in sex and age to the experimental sample was recruited to the present investigation. All patients were selected, evaluated and treated with this robotic technology at Brain Damage Unit (BDU) in Hospital Beata Maria Ana (HBMA). Data have been collected in a prospectively maintained database approved by the hospital review board. Of the 401 patients admitted to HBMA with variable etiology between 2013 and 2018, 241 subjects were evaluated for eligibility, based on compliance with the inclusion criteria: (a) etiology of the current event: stroke. Additionally, they had to meet: (b) first event without recurrence; (b) age between 18 and 80 years; (c) without condition based on sex, man or woman; (d) with neglect or apraxia; (e) with or without activation in the baseline hand; (f) evolution time > 1 month and <3 months (to isolate the “spontaneous recovery” variable and be in the subacute phase of evolution). The exclusion criteria were: (a) cases with clinical instability; (b) presence of active infections that required special respiratory or contact isolation conditions; (c) unconsolidated trauma injuries or with open wounds on the hand or fingers; (d) severe visual deficit; (e) low level of consciousness; (f) severe affectation in cognitive, comprehension and/or behavioral processes; (g) spasticity > 3 on the Ashworth Scale. The demographic and clinical characteristics of both groups are shown in Figure 2.

Allocation to experimental groups defined ad hoc: For the screening of the presence or absence of severe cognitive deficits, the Mini Mental State Examination (MMSE) was used. To determine cognitive problems associated with the hemispheric laterality of the brain injury, the Rivermead Perceptual Assessment Battery (RPAB) and the Florida Apraxia Test (TAF) were administered. Additionally, diagnosis was supported by neurological and neuropsychological clinical criteria. All patients were grouped according to the scores obtained on said scales and the clinical criteria of presence or absence of negligence or apraxia in right and left hemispheric lesions, respectively. The remaining 130 patients who completed the initial clinical screening were randomly assigned to 2 different clinical teams. The 64 subjects finally assigned to team 1 were grouped according to a clinical criterion for convenience, into 2 groups with an ad hoc prognostic profile, one positive (PPP, *n* = 32) and the other negative (PPN, *n* = 32) based on the degree of paresis objectified at admission in the baseline(presence or absence of sensorimotor behavior in finger and wrist activation and/or preservation of sensibility) from the neuromotor and biomechanical muscle functional assessment from neurologist, physician and occupational therapist. A summary of items from the Rivermead Assessment of Somatosensory Performance (RASP) and the Chedoke McMaster Stroke Assessment (CMSA) were used to determine the degree of sensory preservation and finger and/or wrist paresis and grouping in relation to this criteria [24,25,26]. In phase 1, assignment to the initial RAT or COHT intervention group was performed using a computerized program that generated random assignments (Wichmann-Hill Random Number Algorithm), which applied the Lehemer algorithm to equally assign control (COHT) and experimental groups (TAR). In phase 2, each patient received the remaining treatment. Six subjects did not complete all phases of the study and were assumed to have experimental mortality (9.4%), as a consequence of episodes of clinical instability (*n* = 3) or due to loss of follow-up in the control (*n* = 1) or experimental phase (*n* = 2). A total of 58 patients completed the study.

### 2.3. Procedures and Interventions

Parallel to the trial, a systematic review with meta-analysis was carried out last 2 years of the study to determine the level of evidence and the degree of recommendation for the use of the AHR device [1,16].

Assessment protocol: On the first day of the assessment, participants were informed verbally and in writing about study procedures. After signing the informed consent form, they were asked to complete sociodemographic and research questionnaires. All assessment sessions were performed by blinded professionals to the treatment process between groups and experienced occupational therapists in neurorehabilitation and robotics in two different sessions: (i) an initial interview and standardized upper extremity test, and (ii) a computerized test using the AHR kinetic-kinematic tasks.

Measuring instruments: Based on the latest systematic reviews of neurological diseases [9,27,28], primary outcome measures were Fugl-Meyer Assessment for Upper Limb (FMUL) and those derived from the robotic assessment protocol with AHR: active range of motion (ROM Assessment) and grip strength (Force Assessment). Secondary outcome measures were the Nine Hole Peg Test (NHPT), for the evaluation of fine motor function of the hand and digital pinch, the Box and Block Test (BBT), for the evaluation of gross motor function of grip-reach-hand carry and the Assessment of Motor and Process Skills (AMPS), for the evaluation of motor and processing skills involved in the contextualized performance of standardized functional tasks. Repeated outcome measures were obtained at baseline (T1) and completion of each treatment (T2) for each of the phases (T1A, T2A, T1B, T2B).The ad hoc PPP-PPN prognostic profiles were defined as independent factors under study, as well as the laterality-cognition factor associated with each cerebral hemisphere (RH-LH). The kinematic (range of motion) and kinetic (flexor and extensor strength) biomechanical parameters of the AHR device, as well as the scores obtained on the sensorimotor scales (FMUL, BBT and NHPT) and functional (AMPS), were defined as dependent variables and repeated measures of pre-post results.

Intervention protocol: COHT and RAT techniques were selected based on the best level of evidence found, as well as adapted and adjusted for each particular case, seeking the maximum therapeutic benefit in each treatment phase. Specific hand occupational therapy techniques (constraint induced motor therapy, task-oriented motor relearning, mirror therapy, action-observation therapy, motor imaginary, biomechanical analysis, motor execution therapy, neuromotor control approach) were implemented in the COHT control intervention phase, according to therapeutic needs at the discretion of the expert professional. Continuous passive and continuous passive plus robotic assisted therapy programs (CPM and CPMplus), active therapy (AT), as well as interactive therapy (IT) according to the individualized needs of each case, were implemented in the RAT experimental phase, promoting motor learning with AHR version 5, through the integration into therapy of the concepts of intensive and high-frequency repetition of engrams, of increased intrinsic and extrinsic feedback, as well as the possibility of working on motor feed-forward [29,30,31,32,33,34,35]. All patients received this cross intervention (AB-BA) without prejudice to their usual physiotherapy treatment, based on axial control, rehabilitation of the lower limb, posture, stability and balance, in a frequency of 4.5 ± 0.5 weekly sessions during the 6 months of the study, as well as adjuvant speech therapy and neuropsychology sessions at the same average frequency.

### 2.4. Ethics

The present study was approved, from its pre-experimental pilot phase, by the Ethic and Health Care Committee of the Hermanas Hospitalarias Hospital Beata Maria Ana at 2013 (ref. 03/2013). All participants signed the Informed Consent. During the process, ethical principles for medical research were accomplished (Seoul, Korea, October 2008). This study was completed in compliance with institutional research standards for human research and in accordance with the Declaration of Helsinki. REH and MAP^®^ project (ref. 2022A36003) was approved by the Ethic Committee of the Centro Superior de EstudiosUniversitarios La Salle (CSEULS) at October 2020 (ref. PI-040/2020) to give continuity to this research in a post-doctoral phase.

### 2.5. Statistical Analysis

The *sample size* was calculated based on data from a pilot study (*n* = 12) using G*Power 3.1 (University of Düsseldorf, Germany) [32]. The required sample size to detect differences between groups was 51 subjects in total, with an effect size of 0.45, power of 80% and α of 5%. Due to the planned regression analysis (10 subjects per variable), the number of cases was increased to *n* = 60 in order to obtain stable estimates for the regression coefficients [36]. The sample size of the control group of healthy volunteer subjects was matched on study variables (*n* = 30). The data analysis was performed using IBM SPSS (v.27.0; SPSS, Inc., Chicago, IL, USA) statistical software. Shapiro–Wilk test (N < 50 per group) was carried out to test for normal distribution of the data. Descriptive data were presented as mean and standard deviation for quantitative variables, and frequency and percentage for categorical variables.

Comparisons between groups. Variables related to sensorimotor or functional hand recovery were compared between both ad hoc factor groups (on-set prognosis and lateralization of lesion) using a one way analysis of the variance (ANOVA) with Bonferroni-corrected post hoc test. Assumptions of Kolmogorov–Smirnov normality and Mauchly/Greenhouse-Geiser sphericity are assumed in all contrasts. The effect size was calculated using partial eta-squared (η^2^) from the ANOVA analysis [η^2^ between (0.01–0.039), (0.06–0.11), and >0.14 was considered as small, medium or large, respectively] [37]. A repeated-measures ANOVA was carried out to analyse differences in on-set prognosis (PPP or PPN) and lateralization of lesion (RH or LH), considering “sensorimotor” and “functional” as the within factors and “group” as between factors. In case of significant interaction effects, Bonferroni corrected posthoc *t*-tests were performed. All *p*-values are presented with Bonferroni correction. The level of the significance was set at *p* < 0.05. Observed Power statistics (1-β) are provided for each contrast.

Multiple linear regression. Considering that all the variables showed a normal distribution, Pearson’s rank correlation coefficient was used to analyse correlation between dependent and independent variables. Scores range from −1 to 1, with values less than 0.3 meaning weak correlation; 0.3 to 0.7, moderate correlation; and more than 0.7 strong correlation [38]. According to correlation test results, only the three independent variables with highest values of Pearson’s coefficient were entered into a predictive model [39,40]. Multiple backward linear regression models were used to examine the capacity of kinetic and kinematic AHR variables, evaluated during the first session, to predict the variability of the sensorimotor and functional recovery at 3 and 6 months after event. The strength of the association was determined using coefficient (B), R^2^, adjusted R^2^ and *p*-values. Additionally, standardized beta coefficients (β) were included for all predictors in the final model, AMADEUS-Global, to allow direct comparisons between different predictor variables. The regression models were fitted to the entire data set. The level of the significance was set at *p* < 0.10.

## 3. Results

### 3.1. Outcomes between Subacute Stroke Experimental and Healthy Control Groups

Previous analyzes of the literature revealed that there was no history of validation studies of the evaluation tests of the Amadeo^®^ robot in a control group of healthy volunteer subjects [23]. During 2020–2021, both kinetic-kinematic evaluation measures of the robot and the sensorimotor tests included in this research were administered to a control group of healthy volunteers (*n* = 30) matched in sex (60% male, 40% female) and age (58.5 ± 9 years old) to the experimental sample of the present investigation. Statistically significant differences were found in relation to the control group of healthy volunteer subjects (*p* < 0.001) compared to the 4 subgroups investigated by factor (PPP-PPN; RH-LH), both in the biomechanical measurements of the hand robot (AM, FF, EF), as well as in the UL sensorimotor tests (FMUL, BBT, NHPT) and the AMPS functional measure (*p* < 0.001).

### 3.2. Changes between Groups at the End of the Treatment per Factors

#### 3.2.1. Factor 1 Ad Hoc: Severity of Sensorimotor Involvement at Admission

(a) Kinetic and kinematic results. All statistical results can be seen in Table 1.

**Figure 3:**
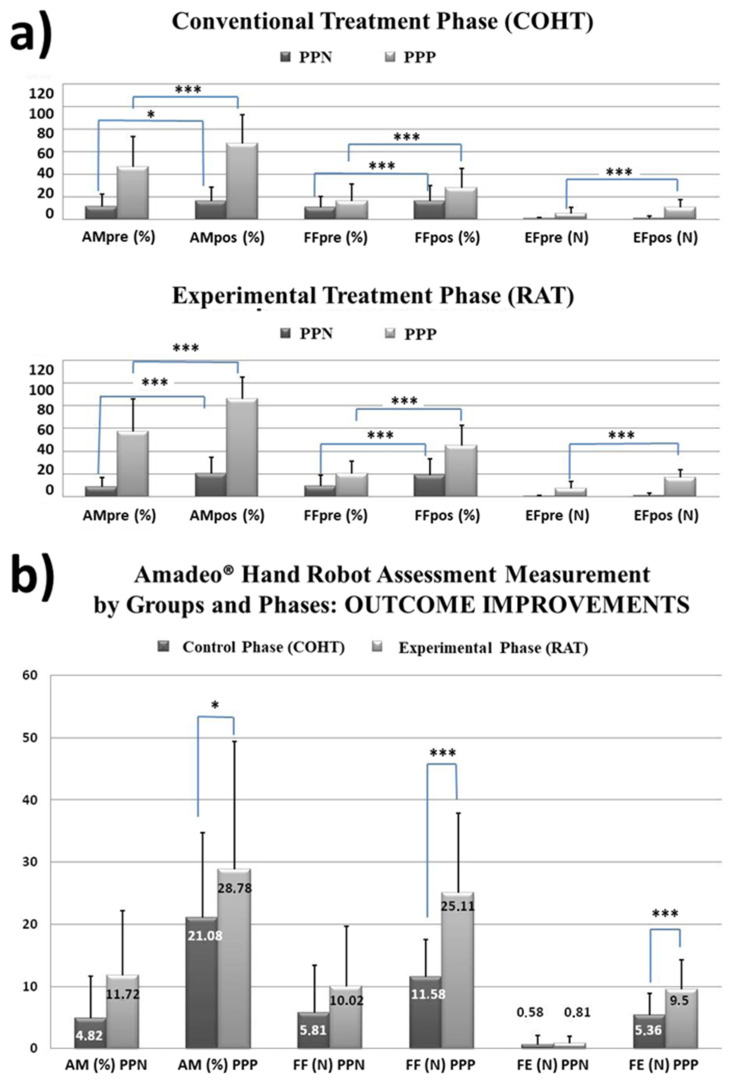
Kinetic and kinematic parameters of the Amadeo^®^ robot at the baseline and at 3 months (**a**) For both ad hoc prognosis groups, the selected treatments, COHT and RAT, had a similar significant effect for the measured variables. Significance levels for the improvements achieved by group and by treatment phase in the variables measured with Amadeo^®^ (**b**). Only the PPP group showed statistically significant differences between treatments for the variables AM (*p* < 0.05), FF (*p* < 0.001) and EF (*p* < 0.001). AM: active range of motion; FF: grip flexor strength; EF: extension strength; N: Newton’s of force; %: = percentage of amplitude; PPN: negative prognostic profile group; PPP: positive prognostic profile group.* *p* < 0.05, *** *p* < 0.001.

The results of the variance factorial design revealed a very significant effect of the *COHT treatment* (control phase) for the AM kinematic parameter (F(1,56) = 83.79, *p* < 0.001, η^2^ = 0.6, OP = 1), thus as for the kinetic parameters of FF (F(1,56) = 93.7, *p* < 0.001, η^2^ = 0.6, OP = 1) and EF (F(1,56) = 67.24, *p* < 0.001, η^2^ = 0.5, OP = 1) measured with the robot. The inter-group results showed a very significant effect of the group factor in the parameters AM (F(1,56) = 69.15, *p* < 0.001, η^2^ = 0.6, OP = 1), FF (F(1,56) = 5.98, *p* < 0.05, η^2^ = 0.1, OP = 0.7) and EF (F(1,56) = 37.46, *p* < 0.001, η^2^ = 0.4, OP = 1). Post hoc analyzes showed significant changes with treatment in the PPN group in AM (*p* < 0.05), FF (*p* < 0.001), but not in EF (*p* = 0.26), while the PPP group showed very significant changes with treatment in AM, FF and EF (*p* < 0.001) (see Figure 3). The analysis of the *RAT treatment* (experimental phase) revealed a highly significant effect for the AM kinematic parameter (F(1,56) = 88.87, *p* < 0.001, η^2^ = 0.6, OP = 1), as well as for the kinetic parameters of FF (F(1,56) = 139.36, *p* < 0.001, η^2^ = 0.7, OP = 1) and EF (F(1,56) = 124.7, *p* < 0.001, η^2^ = 0.7, OP = 1) measured with the robot. The inter-group results showed a very significant effect of the group factor in AM (F(1,56) = 153.1, *p* < 0.001, η^2^ = 0.7, OP = 1), FF (F(1,56) = 33.26, *p* < 0.001, η^2^ = 0.4, OP = 1) and EF (F(1,56) = 83.23, *p* < 0.001, η^2^ = 0.6, OP = 1). Post hoc analyzes showed very significant changes in AM (F(1,56) = 14.88, *p* < 0.001, η^2^ = 0.2, OP = 0.97) and FF (F(1,56) = 22.68 *p* < 0.001, η^2^ = 0.3, OP = 1)for the PPN group, but not in EF (F(1,56) = 1.55, *p* > 0.05, η^2^ = 0.03, OP = 0.2), compared to the group with PPP that shows very significant changes in AM, FF and FE (*p* < 0.001, η^2^ = 0.6–0.8, OP = 1).When analyzing the isolated effect of each treatment by group, the ANOVA statistic showed significant differences between the effect of the conventional treatment A (COHT) and the experimental one B with robotics (RAT), objectifiable in the kinematic parameter of AM (F(1,56) = 7.78, *p* = 0.007, η^2^ = 0.1, OP = 0.8) and in the kinetic parameters of FF (F(1,56) = 30.5, *p* < 0.001, η^2^ = 0.4, OP = 1) and EF (F(1,56) = 15.96, *p* < 0.001, η^2^ = 0.22, OP = 0.97) measured with the robot. Their posthoc analysis showed that only in the PPP group there were significant differences in the treatment effect in favor of robotics (see Figure 3), evidenced in the improvements achieved in the AM (*p* = 0.042), FF (*p* < 0.001) and EF (*p* < 0.001). For the PPN group, both treatments behaved with the same effect.

(b) Functional sensorimotor results. Statistical results can be seen in Table 2

**Figure 4:**
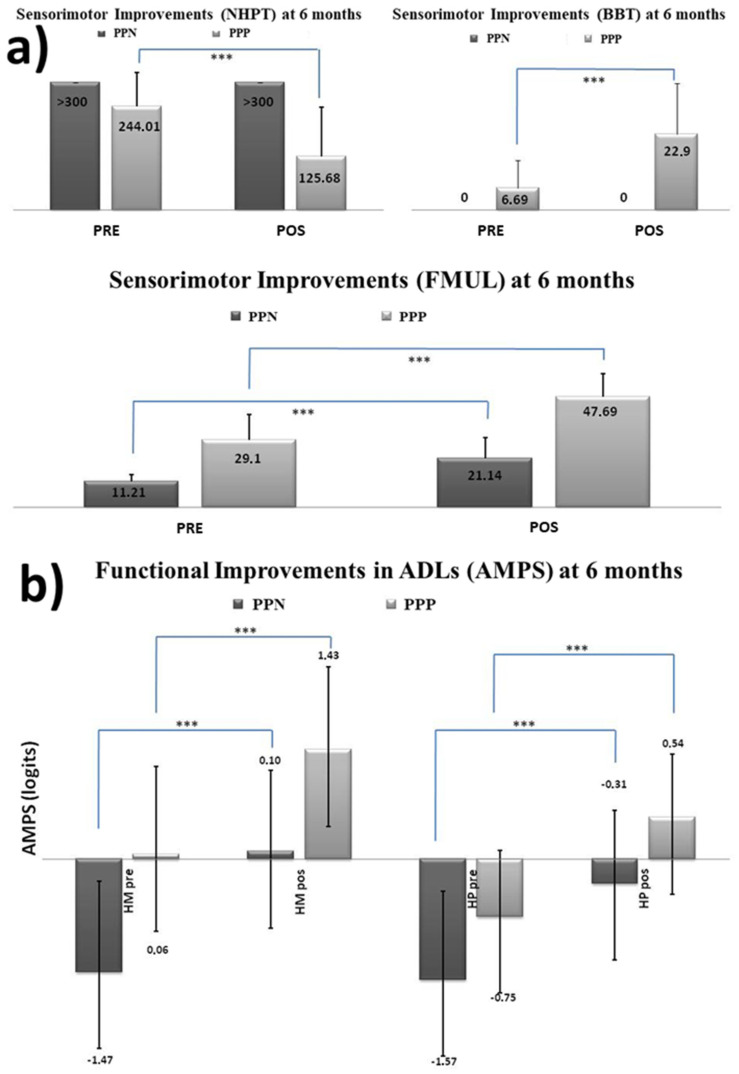
Sensorimotor (**a**) and functional (**b**) effects of the synergistic treatment (i.e., COHT + RAT) at 6 months in the outcome variables NHPT, BBT, FMUL and AMPS©. The FMUL scale showed a significant effect of the combined treatment for both prognosis groups (*** *p* < 0.001), while NHPT and BBT only detected it in the PPP group (*** *p* < 0.001).

The ANOVA analysis showed that the *RAT treatment* at 3 months had a very significant effect on the affected UL sensorimotor improvement measured with the FMUL in both the PPP group (F(1,55) = 194.97, *p* < 0.001, η^2^ = 0.8, OP = 1) as in the PPN group (F(1,55) = 51.07, *p* < 0.001, η^2^ = 0.5, OP = 1). For the BBT (F(1,56) = 114.99, *p* < 0.001, η^2^ = 0.7, OP = 1) and NHPT (F(1,56) = 81.47, *p* < 0.001, η^2^ = 0.6, OP = 1) only statistically significant differences were found in the PPP group. In the PPN group, no statistically significant effect was found (*p* > 0.05). The COHT treatment at 3 months had a very significant effect on the affected UL sensorimotor improvement measured with the FMUL for both the PPP group (F(1,55) = 218.78, *p* < 0.001, η^2^ = 0.8, OP = 1) as for the PPN group (F(1,55) = 71.77, *p* < 0.001, η^2^ = 0.6, OP = 1). For the BBT (F(1,56) = 69.58, *p* < 0.001, η^2^ = 0.6, OP = 1) and NHPT (F(1,56) = 91.02, *p* < 0.001, η^2^ = 0.6, OP = 1) a positive treatment effect was found in the PPP group, with no statistically significant effect being found for the PPN group (*p* > 0.05). The ANOVA analysis showed that the synergistic treatment *(COHT+RAT)* at 6 months (AB or BA) had a very significant effect on the sensorimotor improvement of the affected UL (see Figure 4) measured with the FMUL (F(1,55) = 259.48, *p* < 0.001, η^2^ = 0.8, OP = 1), BBT (F(1,56) = 67.52, *p* < 0.001, η^2^ = 0.5, OP = 1) and NHPT (F(1,56) = 43.94, *p* < 0.001, η^2^ = 0.4, OP = 1), the effect at the inter-group level between patients with PPP and PPN being also significant for the 3 scales (*p* < 0.001). Post hoc analyzes showed a significant effect for FMUL for both the PPP group (F(1,56) = 220.44, *p* < 0.001, η^2^ = 0.8, OP = 1) and PPN group (F(1,56) = 62.94, *p* < 0.001, η^2^ = 0.5, OP = 1). The effect was also significant for the PPP group with BBT (F(1,56) = 87.88, *p* < 0.001, η^2^ = 0.6, OP = 1) and NHPT (F(1,56) = 135.04, *p* < 0.001, η^2^ = 0.7, OP = 1). In contrast, analysis of variance showed no significant changes for the PPN group at 6 months with NHPT (*p* = 0.7) and BBT (*p* = 0.5).

(c) Functional performance in ADL. Statistics are shown in Table 3

The analysis of variance showed that *RAT treatment* at 3 months had a highly significant effect on the performance of activities of daily living in the MS-AMPS motor measure for both the PPP group (F(1,55) = 208.04, *p* < 0.001, η^2^ = 0.8, OP = 1) and the PPN group (F(1,56) = 61.53, *p* < 0.001, η^2^ = 0.5, OP = 1), as in the processing measure HP-AMPS for the PPP group (F(1,55) = 116.38, *p* < 0.001, η^2^ = 0.7, OP = 1) as well as for the PPN group (F(1,56) = 121.41, *p* < 0.001, η^2^ = 0.7, OP = 1). The *COHT treatment* at 3 months had a very significant effect on both the performance of activities of daily living and the HM-AMPS motor measure for PPP group (F(1,55) = 421.09, *p* < 0.001, η^2^ = 0.9, OP = 1) and PPN group (F(1,56) = 132.72, *p* < 0.001, η^2^ = 0.7, OP = 1) as well as in the HP-AMPS processing measure for the PPP group (F(1,55) = 150.7, *p* < 0.001, η^2^ = 0.7, OP = 1) and for the PPN group (F(1,56) = 127.8, *p* < 0.001, η^2^ = 0.7, OP = 1).The post hoc analysis of the *improvements found in each isolated phase* revealed the benefit of RAT treatment versus COHT intervention to increase the measurement in HM-AMPS, both for the PPP group (F(1,55) = 45.1, *p* < 0.001, η^2^ = 0.5, OP = 1) and for the PPN group (F(1,55) = 5.01, *p* < 0.001, η^2^ = 0.3, OP = 1). However, based on the results analyzed, both treatments would have the same therapeutic effect to improve cognition measurable through the HP-AMPS measure (*p* < 0.05). Statistical analysis showed a highly significant effect on the improvements found in ADL performance by applying this synergistic treatment model (AB or BA) at 6 months objectifiable in the HP-AMPS (F(1,55) = 37.72, *p* < 0.001, η^2^ = 0.4, OP = 1) and HM-AMPS measure (F(1,56) = 281.10, *p* < 0.001, η^2^ = 0.8, OP = 1), with a significant inter-group effect between patients with PPP and PPN, both in HP-AMPS (*p* < 0.01) and HM-AMPS (*p* < 0.001). Post hoc analyzes reveal a highly statistically significant effect of synergistic treatment for both the PPP group and the PPN group in HP-AMPS (*p* < 0.001) and HM-AMPS (*p* < 0.001).

#### 3.2.2. Factor 2 Ad Hoc: Laterality of Brain Injury and Its Cognition

(a) Kinetic and kinematic results. All statistical results can be seen in Table 1.

The results of the ANOVA revealed a very significant effect of the COHT treatment for the kinematic parameter of AM (F(1,56) = 54.07, *p* < 0.001, η^2^ = 0.5, OP = 1), as well as for the kinetic parameters of FF (F(1,56) = 83.37, *p* < 0.001, η^2^ = 0.6, OP = 1) and EF (F(1,56) = 37.42, *p* < 0.001, η^2^ = 0.4, OP = 1) measured with AHR. The inter-group results showed the absence of a significant effect of the group factor in all parameters AM (F(1,56) = 0.79, *p* = 0.378, η^2^ = 0.014, OP = 0.14), FF (F(1,56) = 2.03, *p* = 0.160, η^2^ = 0.035, OP = 0.3) and EF (F(1,56) = 0.38, *p* = 0.541, η^2^ = 0.007, OP = 0.1). No significant differences being found between right and left strokes with COHT approaches. In relation to the RAT intervention, the factorial analysis results revealed a very significant effect for the parameters AM (F(1,56) = 69.20, *p* < 0.001, η^2^ = 0.55, OP = 1), FF (F(1,56) = 96.18, *p* < 0.001, η^2^ = 0.6, OP = 1) and EF (F(1,56) = 47.86, *p* < 0.001, η^2^ = 0.5, OP = 1) measured with the robot. The inter-group results showed no significant interaction in AM (F(1,56) = 1.16, *p* = 0.287, η^2^ = 0.02, OP = 0.2), FF (F(1,56) = 1.70, *p* = 0.197, η^2^ = 0.03, OP = 0.25) and EF (F(1,56) = 0.27, *p* = 0.595, η^2^ = 0.005, OP = 0.1). No significant differences being found between right and left strokes with RAT approach.The results of the ANOVA factorial analysis comparing the *improvements* reached in these variables revealed a highly significant effect in favor of RAT for the kinematic parameter of AM (F(1,56) = 7.48, *p* = 0.008, η^2^ = 0.12, OP = 0.8), as well as for the kinetic parameters of FF (F(1,56) = 27.11, *p* < 0.001, η^2^ = 0.33, OP = 0.99) and EF (F(1,56) = 12.86, *p* < 0.001, η^2^ = 0.2, OP = 0.94) measured with the robot. Inter-group results using post hoc contrasts showed a highly significant effect in favor of RAT in left hemiplegic patients in parameters AM (F(1,56) = 10.21, *p* = 0.002, η^2^ = 0.2, OP = 0.9), FF (F(1,56) = 29.68, *p* = 0.000, η^2^ = 0.3, OP = 1) and EF (F(1,56) = 7.55, *p* = 0.008, η^2^ = 0.3, OP = 1), while for right hemiplegic patients only statistically significant differences were found in FF (F(1,56) = 4.15, *p* = 0.46, η^2^ = 0.1, OP = 0.5) and EF (F(1,56) = 5.48, *p* = 0.023, η^2^ = 0.1, OP = 0.6), but not in AM (F(1,56) = 0.574, *p* = 0.45, η^2^ = 0.01, OP = 0.1).Considering the improvements and not the scores, the RAT is more beneficial for right hemispheric patients with neglect.

(b) Functional sensorimotor results (See Table 2)

The results of analysis of variance revealed a highly significant effect of the *COHT treatment* on the FMUL upper limb global functionality scale (F(1,56) = 222.03, *p* < 0.001, η^2^ = 0.8, OP = 1), on the BBT gross manual dexterity scale (F(1,56) = 22.52, *p* < 0.001, η^2^ = 0.3, OP = 0.99) and on the NHPT fine manual dexterity scale (F(1, 56) = 24.84, *p* < 0.001, η^2^ = 0.3, OP = 0.99) for both groups of hemispheric involvement. The inter-group results did not show significantdifferences associated with the group factor in NHPT (F(1,56) = 0.69, *p* = 0.794, η^2^ = 0.001, OP = 0.1) and BBT (F(1,56) = 0.20, *p* = 0.655, η^2^ = 0.004, OP = 0.1), but it was in the FMUL (F(1,56) = 5.30, *p* = 0.025, η^2^ = 0.086, OP = 0.62). The Bonferroni post hoc tests showstatistically significant differences in pre-post test measures (*p* < 0.01) in favor of right hemispheric lesions. In relation to the *RAT intervention*, the results of ANOVA revealed a very significant effect of the treatment for the FMUL global functionality scale (1.56) = 164.8, *p* < 0.001, η^2^ = 0.75, OP = 1), for the BBT gross manual dexterity scale (F(1,56) = 27.8, *p* < 0.001, η^2^ = 0.3, OP = 0.99), as well as for the NHPT fine manual dexterity scale (F(1,56) = 23.71, *p* < 0.001, η^2^ = 0.3, OP = 0.99). The inter-group results found no significant differences associated with the laterality-cognition factor for any of the scales (*p* > 0.05, η^2^ = 0.02–0.034, OP = 0.06–0.28). The results of factorial analysis that compare the *sensorimotor improvements* show a very significant effect in favor of RAT with Amadeo^®^ for the FMUL (F(1,56) = 53.61, *p* < 0.001, η^2^ = 0.49, OP = 1), BBT (F(1,56) = 9.39, *p* = 0.003, η^2^ = 0.14, OP = 0.85) and NHPT (F(1,56) = 16.96, *p* < 0.001, η^2^ = 0.23, OP = 0.98). However, the inter-group results did not show a more significant effect in either of the two groups compared to the other, associated with the laterality-cognition factor (*p* > 0.05, η^2^ = 0–0.02, OP = 0.05–0.18).

(c) Functional performance in activities of daily living (see Table 3)

The results revealed a very significant effect of the COHT treatment in the AMPS©measure of functional performance in ADL, in the assessment of competence in motor skills HM-AMPS (F(1,56) = 302.08, *p* < 0.001, η^2^ = 0.84, OP = 1), although no significant differences were found between groups related to the hemispheric laterality of the lesion (F(1,56) = 0.69, *p* < 0.41, η^2^ = 0.012, OP = 0.13). In relation to HP-AMPS processing skills, a very significant effect was found (F(1,56) = 274.61, *p* < 0.001, η^2^ = 0.83, OP = 1), although the inter-group did not detect statistically significant differences associated with left paresis versus right paresis (F(1,56) = 0.06, *p* < 0.94, η^2^ = 0.0, OP = 0.05). In relation to the *RAT intervention* the results of the factor analysis of variance revealed a very significant effect on HM-AMPS motor skills (F(1,56) = 179.2, *p* < 0.001, η^2^ = 0.76, OP = 1), although the inter-group effect did not detect statistically significant differences associated with hand paresis with apraxia versus hand paresis with neglect (F(1,56) = 0.15, *p* < 0.69, η^2^ = 0.003, OP = 0.1). In relation to the HP-AMPS processing skills, a very significant effect of the intervention was found (F(1,56) = 236.25, *p* < 0.001, η^2^ = 0.81, OP = 1), although the inter-group effect neither was detected (F(1,56) = 0.07, *p* < 0.78, η^2^ = 0.001, OP = 0.06). Bonferroni post hoc analyzes showed highly statistically significant differences in HM and HP-AMPS test–retest for both RH/LH groups (*p* < 0.001) after applying both traditional hand therapy and robotic therapy with Amadeo^®^, without the effect being greater in either group.The results comparing the improvements found in the HM-AMPS in COHT phase versus the RAT phase revealed a highly significant treatment effect in favor of RAT (F(1,56) = 34.27, *p* = < 0.001, η^2^ = 0.38, OP = 1), although no differences were found in the interaction (F(1,56) = 0.16, *p* = 0.69, η^2^ = 0.003, OP = 0.1). In relation to the HP-AMPS measurement, no significant effect of the intervention was found (F(1,56) = 1.16, *p* = 0.286, η^2^ = 0.02, OP = 0.2) neither its interaction in function of the factor laterality-cognition (F(1,56) = 2.36, *p* = 0.13, η^2^ = 0.04, OP = 0.3).The results that analyze the effects of a synergistic intervention at 6 months (COHT+RAT) show for HM-AMPS a highly statistically significant effect for treatment (F(1,56) = 240.39, *p* < 0.001, η^2^ = 0.8, OP = 1), although no differences were found in the interaction(F(1,56) = 0.331, *p* = 0.57, η^2^ = 0.006, OP = 0.1). A very significant effect of the treatment was found for HP-AMPS (F(1,56) = 353.45, *p* < 0.001, η^2^ = 0.9, OP = 1) but not in its interaction (F(1,56) = 0.019, *p* = 0.89, η^2^ = 0.00, OP = 0.05).The Bonferroni post hoc analyzes showed highly statistically significant differences for the adjutant intervention in the HM and HP-AMPS measures for both RH/LH groups (*p* < 0.001).

### 3.3. Outcome Clinical Predictive Model with Amadeo Hand Robot

A total of 18 linear regression models were analyzed, discarding those whose criteria variables or factors did not meet the requirements of normal distribution or homoscedasticity. Those variables that showed co-linearity and multi-co-linearity were also discarded. The results of the analysis of the dependency relationship between factors, criteria variables and potentially predictors are shown in Table 4. A moderate to strong dependency relationship is found between the variables subjected to modeling in the Pearson´s correlation coefficient, from 0.4 to 0.8, with a significance level of *p* < 0.000. Ultimately, two predictive models, Amadeus-A and Amadeus-B, have been generated to, through the improvements inevaluation parameters of the Amadeo^®^ robot (AM, FF and EF) from first session day (*1d), be able to predict the functional changes in the AMPS tool, as well as the sensorimotor changes in the FMUL, 9HPT and BBT scales, at 3 and 6 months after the intervention, regardless of whether it is conventional (COHT), with robotics (RAT) or in synergy (COHT + RAT). Each improvement variable (*1d) of the robot’s AM, FF and EF parameters has been obtained from the intra-session pre-post measurements, obtained on the first day of intervention (for example, AM*1d = AM post -AM pre). The Amadeus-A regression models for the functional criteria variables HMAmps_3months and HMAmps_6months, as well as the Amadeus-B regression model for the sensorimotor criteria variables FMUL_6months, BBT_6months, and NHPT_6months are presented in Table 5.

#### 3.3.1. Functional ADLs Outcome Predictive Model

The Amadeus-A1 sub-model, based on the independent variable AM and FF, explains 51% of the variance in the dependent variable that measures the improvement of motor skills in the AMPS scale measured at 3 months of intervention with COHT intervention. The Amadeus-A2 model, through the predictor variable EF, explains 34% of the variance of the improvements found in the AMPS tool measured at 3 months of intervention with a RAT intervention with Amadeo^®^ robot. The model Amadeus-A3, based on independent variable AM and EF, predicts behavior at 43% of the criteria variable that measures improvement in AMPS motor skills at 6 months with a synergistic intervention (COHT+RAT).

#### 3.3.2. Sensorimotor Outcome Predictive Model

The Amadeus-B1 sub-model, based on the independent variables FF and EF, shows covariance to predict 64% of the variability in the behavior of the global motor functionality of the upper limb with FMUL at 6 months of synergistic intervention (COHT+RAT). The Amadeus-B2 sub-model, using the same variables, is able to explain 62% of the variance in the gross manual dexterity with BBT after 6 months of robotic and conventional treatment. The Amadeus-B3 sub-model, using the same predictor variables, explains 65% of the variance in the fine manual dexterity with the sensorimotor scale NHPT at 6 months of synergistic intervention.

The analysis of the behavior between factors was carried out, as well as the estimation of the strength of association between criterion and predictor variables in the regression models. The AM kinematic variable of the AHR device was identified as a significant covariate along with FF for predicting changes in AMPS motor skills at 3 months of receiving COHT (adjusted R^2^ = 0.51; β = 0.41; *p* = 0.001) and for predicting changes in AMPS motor skills 6 months after receiving a synergistic intervention (adjusted R^2^ = 0.43; β = 0.31; *p* = 0.012). AM turned out to be a predictor variable of functional recovery in 2/3 AMADEUS-A sub-models, but it was eliminated for the AMADEUS-B sensorimotor recovery prediction models. For the rest of the sensorimotor and functional sub-models it was eliminated (4/6). In relation to the FF kinetic variable of the AHR device, the regression model shows that a combination of FF and AM were significant predictors for the recovery of functionality in AMPS motor skills at 3 months of receiving COHT (adjusted R^2^ = 0.51; β = 0.39, *p* = 0.001). A combination of FF and EF were able to explain 64% of the variance of the improvements found in the FMUL upper limb global functionality scale scores (adjusted R^2^ = 0.64; β = 0.29; *p* = 0.004), on 62% of the variance of the improvements found in the BBT gross motor skill scale scores (adjusted R^2^ = 0.62; β = 0.23; *p* = 0.028) and 65% of the variance of the improvements found in the NHPT fine motor skills scale scores (adjusted R^2^ = 0.65; β = −0.27; *p* = 0.007). FF turned out to be a predictor variable for functional recovery in 1/3 AMADEUS-A sub-models and 3/3 AMADEUS-B sub-models, being eliminated only for 2/3 of the functional recovery prediction sub-models. Finally, in relation to the EF kinetic variable of the AHR device, the regression model shows that the EF variable was capable of predicting 34% of the variance of the recovery of functionality in AMPS motor skills at 3 months after receiving an intervention. RAT (adjusted R^2^ = 0.34; β = 0.59; *p* < 0.001). This predictive variable, in combination with the AM variable, were able to predict the behavior of the variance in 43% of the criterion variable related to the recovery of functionality in AMPS motor skills at 6 months after receiving a synergistic intervention (adjusted R^2^ = 0.43, β = 0.45, *p* < 0.001). A combination of EF and FF were able to explain 64% of the variance of the improvements found in the FMUL scale scores (adjusted R^2^ = 0.64; β = 0.60; *p* < 0.001), 62% of the variance of the improvements found in BBT scores (adjusted R^2^ = 0.62; β = 0.65; *p* < 0.001) and 65% variance of improvements found in NHPT scores (adjusted R^2^ = 0.65; β = −0.62; *p* < 0.001). EF turned out to be a predictive variable of functional recovery in 2/3 AMADEUS-A sub-models (66%) and in 3/3 AMADEUS-B sub-models (100%), being eliminated only for 1/3 of the prediction sub-models of functional recovery (33%) and in 1/6 of the total predictive models (16.6%). The total participation of the predictive variables on the criterion variables analyzed in the AMADEUS-A and AMADEUS-B models was 33.3% for AM, 66.6% for the FF variable and 83.3% for the EF variable. Therefore, this last predictive variable is considered the most important.

The validity analysis of each AMADEUS linear regression predictive sub-model was based on compliance with the assumptions of normal distribution in the residuals and homoscedasticity in the variances of the residuals, through new analyzes of the standardized residuals and a diagram of dispersion type scatter plot that allowed evaluating the variance around the residuals. Cook’s distances (D) to identify the influence of possible outliers in the regression model showed that the values were not influential as they were all below D < 1 (D = 0.12 to 0.49). Through linear regression G-G graphs (with means, standard deviations and outliers) and their corresponding regression lines, the possible influence of the outliers on inflation was analyzed, determining it as low and not affecting the predictive accuracy of the model. The auto-correlation assumptions, through the Durbin-Watson statistic, showed independence of the residuals (1.4 to 1.8). Finally, the assumptions of non-multi-co-linearity were analyzed through the variance inflation factor (VIF) and showed values much lower (1.00–1.77) than those representative of high multi-co-linearity (VIF > 10).

## 4. Discussion

In this study, the sensorimotor and functionality in ADLs competence of patients with subacute stroke was analyzed by ad hoc research factors in terms of difficulties and limitations in structures and functions of the paretic hand-UL [41] through the use of the FMUL, BBT, NHPT and AMPS scales. These aspects were assessed after the application of two different therapeutic interventions (i.e., COHT and RAT), and their impact was analyzed in terms of the described outcome variables. Previous studies investigating sensorimotor recovery with the same functional parameters in patients with chronic and sub acute stroke can be found elsewhere [29,31,32,33,34,42,43,44,45,46].

In relation to the first factor analyzed, an important cause-effect association has been found in relation to the degree of paresis diagnosed on admission. Our results could suggested that a distal approach of the upper limb based on COHT techniques could improve the global functionality of the affected arm, stimulating changes in specific motor skills of the hand, which could migrate to other adjacent body segments, improving functions of calibration of grip strength, grasp, transportation, coordination or speed of executionand could improve somatognosis and body awareness, motor control, spatial-body organization and movement timing, reducing maladaptive learned under-use and thus improving integration in the functional context of the affected UL [1,47]. The significant improvements found in kinetic and kinematic parameters denote the existence of different sensorimotor recovery patterns between PPP and PPN groups, as well as a greater treatment benefit for the ad hoc PPP group. Both prognosis profiles could be identified with the AHR device [16]. The presence of greater brachial involvement due to flaccid paralysis, severe paresis or absence of activation in the wrist and/or fingers, typical in PPN, could condition the results towards a worse sensorimotor recovery at 3 months of intervention. On the contrary, a PPP could lead to faster and more significant improvements in a shorter intervention time. The RAT effects found have turned out to be very significant for both prognostic groups. Its implementation allowed to monitor the improvement in kinematic and kinetic parameters of the paretic hand and to detect intra-session and intra-subject positive effects both in the hand and in the fingers [48,49]. Combining the clinical application of robotics with concepts of motor imagery, observation of actions, or motor execution based on neuromotor control, could optimize the process of motor relearning of specific fundamental skills related to amplitude, force, or grip engrams, related to the motor sequencing of the fingers, the temporality of selective and independent movement or the calibrated control of force [6,7,16,50,51]. In this study, the effects of both treatments, RAT and COHT, showed a similar level of significance for the PPP group at 3 months. The effect of robotics in this group was between 33% and 200% more effective in enhancing biomechanical parameters representative of greater selective activation capacity [49,52]. The effect was slightly greater with RAT for PPN in the AM kinematic variable. Neither of the 2 interventions was shown to be effective in increasing kinetic EF in the PPN group. Only in the PPP group statistically significant differences can be seen between the improvements achieved after 3 months of isolated treatment between both types of approach, considering the effect of COHT and RAT to be similar for the PPN group.

The FMUL scale has been shown to be sensitive for monitoring sensorimotor recovery of the UL-hand regardless of the degree of finger-activation on admission in the ad hoc prognosis groups [27,53]. The time elapsed since the event and the magnitude of motor deficit could be associated with the changes observed in this scale. Nevertheless, BBT and NHPT only detect improvements in the PPP group, being useful tools to detect the absence of recovery in these sensorimotor parameters. Our functional outcome measures, based on the specific methodology of the AMPS© model, have made it possible to detect intra- and inter-subject functional changes in ADLs performance in real context. Significant differences in functional performance in ADL between PPP and PPN groups had been detected. However, 3 months could be a short time to estimate statistically significant changes in the ADLs functional context [7,54,55], although our research had detected changes in motor and process skills with both types of intervention. Based on the analysis of the improvements in the functional variables, a significant effect of the RAT compared to the COHT has been observed in motor skills in both prognostic groups, which has not been identified for the processing skills, despite the existence of studies that describe the positive cognitive effect of this robotic technology [16,43,45,56].

Furthermore, according to the current literature, AHR device could improve the motor control of affected UL or facilitate spontaneous recovery processes to be redirected more efficiently [8,43,44,57,58]. Compared to the prevalence of a chronic deficit of the upper limb of more than 50% of post-stroke cases [12], only 40% of patients with PPN who showed null or poor sensorimotor recovery and less than 10% of subjects showed better recovery. These results suggest the usefulness, efficacy and efficiency of the use of motor residue integration strategies in hand paresis and of the application of compensation strategies in synergy with other synergic restorative models, such as both the RobHand2015 and IS-BRAIN models described [1]. Moreover, regardless of the time of evolution since event, 79% of patients with moderate involvement on admission or 18% of strokes doomed to sensorimotor failure with severe initial involvement, according to our data, have benefited from the intensive UL-hand intervention [26,59]. The use of robotic devices for the hand in clinical practice could increase individual selective activation of some fingers and reduce dysfunctional co-activation of others, improving specific radial or ulnar grasping skills as opposed to pathological synergistic motor patterns [49,60,61].

In relation to the second analyzed factor, both right and left stroke patients can benefit from both types of intervention, RAT or COHT, without objectifying differences associated with the laterality of the injury-cognition factor. Analyzing the isolated effect of the treatments on the improvements achieved in the kinetic-kinematic parameters, the post hoc analysis showed that for people with left hemiplegia, RAT is particularly effective in restoring AM, FF, and EF compared to a more conventional approach such as COHT. In right-sided hemiplegic patients, both treatments have the same effect in restoring AM, with RAT being superior in improving FF and FE kinetic parameters of the hand. In relation to sensorimotor recovery, no differences have been identified between right and left hemiplegics when comparing the different types of intervention and the improvements achieved at 3 and 6 months, so both profiles of patients with typical hand hemiparesis could benefit regardless of both types of approaches, conventional and robotic. Only the FMUL identified differences in sensorimotor recovery between right and left strokes after the COHT phase [44,46].These findings emphasize that the selection of hand rehabilitation approaches should be based on factor 1 and not factor 2, preferably. In addition, although previous studies investigated sensorimotor recovery with the same scales in subacute stroke patients, this is the first research to include the AMPS tool to measure ADL competence and participation in people with hand hemiparesis in subacute stroke after receiving a crossover intervention with robotic and conventional approaches. Nonetheless, no differences were found in ADL performance between right and left hemiplegic groups, so that there are no significant effects in favor of one or the other intervention in relation to the laterality-cognition factor [62,63].

The relevance of cognitive processes has been scarcely studied in research on the application of robotic therapies. Cognition is a very important factor in complex motor relearning processes and should not be ignored in the design of experimental studies with robotic technology, since patients with cognitive deficits are excluded in up to 76% of trials [11]. Optimal cognitive processes would contribute to improving cognitive and motor results, constituting the cognitive reserve, which must be taken into account when applying different RAT or COHT techniques [56]. The presence of apraxia or neglect can alter motor control and relearning processes, and it is necessary to analyze them in specific effectiveness studies for each rehabilitation model, whether with robotic devices or not, since the presence of some of these cognitive alterations atypical, such as anosognosia, somatoparaphrenia or alien hand syndrome could complicate the hand rehabilitation process [1].Furthermore, according to the literature, cognitive improvements can be seen in stroke patients after the application of RAT with Amadeo^®^ device [16,32,43,45,56].

Moreover, a recent multicentre clinical trial carried out on stroke patients reported improvements in sensorimotor and upper limb functionality, but not in ADL performance, with a robotics approach, but without exploring the isolated use of the AHR device, so the true effect of its application is difficult to define [58].

Interestingly, current systematic reviews and meta-analysis [13,15,64] are also in line with previous and novel evidence found in this investigation. Additionally, in line with a recent and novel study based on surface electromyography and this hand device, evidences showing that sEMG strength control parameters in combination with AHR could help predict hand sensorimotor ability with FMUL or MAS [65]. However, no predictive *model* like this one has been found that includes evaluation of ADLs in a real context and not through simulated tasks [22], which increases the added value of this research.

**Figure 5:**
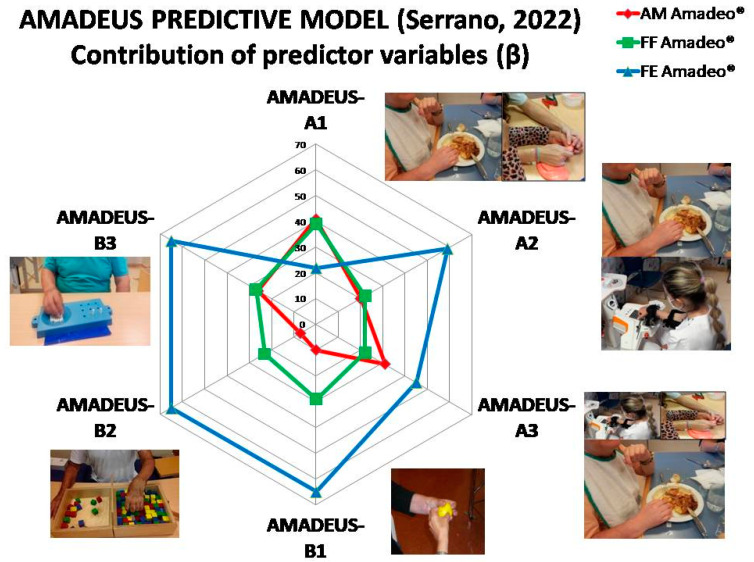
Submodels composing the AMADEUS Global Predictive Model. Contribution of predictor and criteria variables, with regression graphics.

Ours results have therefore implications for clinical practice and future studies. Beyond the changes that we can objectify on sensorimotor and functional behavior in clinical practice, future studies with newer and more complex methodological designs are necessary to determine the true extent of the changes at the level of reactivation due to cortico-subcortical synaptogenesis and brain map reorganization [66,67]. A larger number of studies are still needed that include more objective assessment systems of neurophysiologic changes and true neuroplastic or neurofunctional effects by quantitative electroencephalography (qEEG), transcranial magnetic stimulation (TMS), or functional magnetic resonance imaging (fMRI), by improving the imbalance due to interhemispheric hyper-excitability or by direct or indirect stimulation of complex motor control nuclei, such as primary pre-motor, motor, and somatosensory networks, or supplementary motor area [44,49,68,69,70,71]. Robotic technology like Amadeo^®^ could be used to establish, through its kinetic and kinematic measurement parameters, the possible dose of treatment administered to patients during rehabilitation sessions [13].In the same way, the clinical recommendation that new technologies should not be used massively but implemented by expert professionals individually is reinforced [48]. Not all patients are candidates for robotics. The correct selection and use of robotics will avoid generating false expectations of recovery or frustration during rehabilitation, and will optimize the process of sensorimotor and functional recovery in stroke from the sub-acute phase. Similarly, *adequate prognostic factors* must be used in a clinical context to guarantee the effectiveness and universality of the interventions. In our research, less than 5% of the patients defined a priori for each prognostic group based on their degree of paresis at admission showed a recovery outside the expected prognostic pattern [59,72]. These new technologies will allow us to further increase the objectivity of the evaluation and treatment processes of the upper limb in stroke, not only at the proximal level but also in the distal segment, through the progressive integration of future new theoretical concepts of neurorehabilitation. It would be necessary to allocate more resources for the interdisciplinary development of new technological tools that are closer to the real clinical approach. Focusing our efforts on the recovery of the kinetic-kinematic parameters of the hand through a purposeful intervention, focused on the functional and technical-specific task to enhance the sensorimotor components, would be a safe bet knowing this dependency relationship and the proposed predictive model (see Figure 5). We consider it a milestone for this research to propose the first AMADEUS predictive model of recovery and/or absence of recovery for the typical neurological hand in patients with stroke in the subacute phase for its standardized clinical application with the Amadeo^®^ hand robot. The results found increase the future range of therapeutic and research possibilities with this device, building a bridge towards a clinical application oriented towards other non-neurological or neurological etiological populations, acquired or degenerative.

Some limitations of this study must be noticed. First, although the nature of the methodological design of this research is randomized and controlled, expanding the original pilot study, it is not completely unbiased. Second, despite their robust statistical significance, our results should be interpreted with caution in terms of clinical implementation due to their effect variability and the disparity of clinical signs in stroke patients. New studies with more complex statistical analyzes are necessary to isolate the variables and analyze the effect of co-variables that may condition the results found, as well as to determine the true effect of an intervention with this RAT device on the sensorimotor domain and its impact in functionality. Fourth, the external validity of this model has not been analyzed in depth yet and could be reduced based on the fact that the sample represents a stroke population from a single hospital and geographic area. It is then recommended to reproduce the design in larger and multicenter samples, so that considerations of some potential predictors are not impeded. Similarly, no hold-out or cross-validation approach was used to test the model on unseen data, so the generalizability of our conclusions might be limited. Fifth, the dichotomization of continuous variables should be avoided, as well as limiting the number of predictors included in the models, not to exceed the number of events theoretically estimated and not to overestimate the effects. In this work, an effort has been made to avoid the risk of over-fitting (over-fitting bias), which is why the models have been shown without eliminating the outliers, so that inflation does not affect the predictive accuracy of each model. This may have contributed, however, to the underestimation of some possible true effects. A correct initial analysis of the correlation of the predictor variables, as well as their adjustment to the number of outcome events, made possible to control the risk of predictor selection bias. Sixth, the possible covariate effect of other variables and assessment tools that have not been analyzed is recognized. Seventh, this research is limited to stroke patients in the subacute phase with typical hand hemiparesis, excluding all those atypical cases. Other profiles of vulnerable patients with atypical neurological hand syndromes should be included in future research. Finally, all patients included in this study continued to receive the usual physiotherapy treatment during this trial without prejudice to their planned rehabilitation programs, so the effect of this covariate, although found in both intervention groups, has not been isolated and should be taken into consideration for future explanatory or predictive studies.

## 5. Conclusions

This research focused on the results of a 5-year randomized clinical study with the Amadeo^®^ robot. It demonstrates the added value of this device as a primary assessment and treatment tool in upper limb protocols. Additionally, it proves the usefulness of the innovative clinical predictive AMADEUS model, which complements the IS-BRAIN synergistic, interventionist and theoretical model in our Robotics Unit.

Robot-assisted therapy with Amadeo^®^ significantly improved sensorimotor functionality and manual dexterity in patients with subacute stroke 3 months after the intervention, regardless of the degree of sensorimotor involvement at the beginning of the intervention and the cerebral hemisphere affected by the stroke event. Significant changes in ADL performance could be observed in the AMPS motor skills measurements at 3 months of intervention in patients with greater preservation of hand activation at the admission. Robotic intervention with this hand device and/or conventional hand therapy during 3 to 6 months of treatment improved previous basal sensorimotor and functional results in the four patients described in this research (PPP, PPN, HD, HI).

Finally, the kinetic/kinematic variables of the Amadeo^®^ hand robot (AM, FF and EF), obtained in a first intervention session, can predict, in the subacute phase, functional changes in ADLs at 3 and 6 months of intervention, as well as sensorimotor results at 3 and 6 months in patients with hand hemiparesis due to stroke. Specifically, EF and FF best explain sensorimotor changes, while AM and EF are better predictors of improvements in ADL performance in the real context.

## Figures and Tables

**Table 1:** Kinetic-kinematics effects of COHT and RAT treatments at 3 and 6 months by both prognosis (PPP, PPN) and lateralization-cognition groups (RH, LH).

		Hand Hemiparesis at Admission Factor											Lateralization-Cognition Factor										
Intervention	Kinetic & Kinematic Parameters of Amadeo^®^	PPP Group *n* = 29 M(SD)	F(1,56)	*p* Value	η^2^	OP	PPN Group *n* = 29 M(SD)	F(1,56)	*p* Value	η^2^	OP	Total *N* = 58 M(SD)	RH Group *n* = 27 M(SD)	F(1,56)	*p* Value	η^2^	OP	LH Group *n* = 31 M(SD)	F(1,56)	*p* Value	η^2^	OP	Total *N* = 58 M(SD)
Experimental Treatment (A) RAT (3 months)	AM pre (%) AM pos (%)	57.2(29.2) 86(19.3)	89.75	<0.001 ***	0.6	1	8.4(8.3) 20.1(14.9)	14.88	<0.001 ***	0.2	0.97	32.8(32.5) 53.1(37.3)	29.7(6.3) 47.2(7.2)	24.54	<0.001 ***	0.3	1	35.5(5.7) 58.2(6.7)	47.39	<0.001 ***	0.5	1	32.6(4.3) 52.7(4.9)
	FF pre (N) FF pos (N)	20.3(11.3) 45.4(17.5)	142.38	<0.001 ***	0.7	1	9.1(9.9) 19.1(14.3)	22.68	<0.001 ***	0.3	1	14.7(11.9) 32.3(20.7)	14.6(2.3) 29.7(4)	33.80	<0.001 ***	0.4	1	14.7(2.2) 34.5(3.7)	66.33	<0.001 ***	0.5	1	14.7(1.6) 32.1(2.7)
	EF pre (N) EF pos (N)	7.14(6.37) 16.6(7.4)	211.7	<0.001 ***	0.8	1	0.28(1.03) 1.1(1.9)	1.55	0.22 ns	0.03	0.2	3.7(5.7) 8.9(9.5)	3(1.1) 7.7(1.8)	19.06	<0.001 ***	0.3	1	4.4(1) 9.9(1.7)	29.82	<0.001 ***	0.04	1	3.7(0.8) 8.8(1.3)
Control Treatment (B) COHT (3 months)	AM pre (%) AMpos (%)	46.2(27.1) 67.2(25.7)	111.02	<0.001 ***	0.7	1	11.1(11.1) 15.9(12.7)	5.80	0.019 *	0.1	0.7	28.6(27.1) 41.6(32.8)	25(5.2) 39.6(6.3)	31.77	<0.001 ***	0.4	1	31.8(4.8) 43.3(5.9)	22.45	<0.001 ***	0.3	1	28.4(3.6) 41.5(4.3)
	FF pre (N) FF pos (N)	16.2(14.8) 27.8(17.4)	83.11	<0.001 ***	0.6	1	10.1(10.1) 15.9(14.1)	20.91	<0.001 ***	0.3	1	13.2(12.9) 21.9(16.8)	12.7(2.5) 22.8(3.3)	52.10	<0.001 ***	0.5	1	13.6(2.3) 21(3)	31.90	<0.001 ***	0.4	1	13.1(1.7) 21.9(2.2)
	EF pre (N) EF pos (N)	5(5.4) 10.4(6.9)	109.4	<0.001 ***	0.7	1	0.44(0.89) 1(2.1)	1.29	0.26 ns	0.02	0.2	2.7(4.5) 5.7(6.9)	2.1(.8) 4.8(1.3)	14.16	<0.001 ***	0.2	0.9	3.2(0.8) 6.4(1.2)	24.34	<0.001 ***	0.03	1	2.7(0.6) 5.6(0.9)

Descriptive, F test values with their respective degrees of freedom, effect sizes (η^2^) and statistical power (OP) are shown. AM: active range of motion, FF: grip flexor strength, EF: release extensor strength; %:percentage of AM; N: Newtons of force; COHT: conventional hand-specific occupational therapy; M: mean; SD: standard deviation; PPP: positive prognostic profile subgroup, PPN: negative prognostic profile subgroup; RAT: robot-assisted therapy; F: ANOVA F test with degrees of freedom; ns: not significant; * *p* < 0.05 *** *p* < 0.001.

**Table 2:** Sensorimotor effects of COHT and RAT treatments at 3 and 6 months by both prognosis (PPP, PPN) and lateralization-cognition groups (RH, LH).

		Hand Hemiparesis at Admission Factor											Lateralization-Cognition Factor										
Intervention	Sensorimotor Variables by Scale	PPP Group *n* = 29 M(SD)	F(1,56)	*p* Value	η^2^	OP	PPN Group *n* = 29 M(SD)	F(1,56)	*p* Value	η^2^	OP	Total *N* = 58 M(SD)	RH Group *n* = 27 M(SD)	F(1,56)	*p* Value	η^2^	OP	LH Group *n* = 31 M(SD)	F(1,56)	*p* Value	η^2^	OP	Total *N* = 58 M(SD)
Experimental Treatment (A) RAT (3 months)	FMUL pre FMUL pos	36(1.5) 47.7(1.7)	194.97	<0.001 ***	0.8	1	15.2(1.5) 21.1(1.7)	51.07	<0.001 ***	0.5	1	25.6(1.1) 34.4(1.2)	25.8(2.6) 35.7(3.2)	94.92	<0.001 ***	0.6	1	25.4(2.4) 33.3(3)	70.13	<0.001 ***	0.6	1	25.6(1.8) 34.5(2.2)
	NHPT pre NHPT pos	199(11) 126(15)	81.47	<0.001 ***	0.6	1	300(11) 300(15)	0	1 ns	0	0.05	249(7.7) 213(10.7)	254(15) 215(23)	12.51	<0.01 **	0.2	0.9	245(14) 211(22)	11.20	<0.01 **	0.02	0.9	250(10) 213(16)
	BBT pre BBT pos	12.8(1.4) 22.9(2)	114.99	<0.001 ***	0.7	1	0(1.4) 0(2)	0.001	0.97 ns	0	0.05	6.4(1) 11.4(1.4)	6.5(1.9) 11.2(3)	11.43	<0.01 **	0.2	0.9	6.4(1.8) 11.7(2.9)	16.81	<0.001 ***	0.2	1	6.4(1.3) 11.4(2.1)
Control Treatment (B) COHT (3 months)	FMUL pre FMUL pos	29.1(1.5) 36.2(1.5)	218.78	<0.001 ***	0.8	1	11.2(1.5) 15.3(1.5)	71.77	<0.001 ***	0.6	1	20.2(1) 25.7(1.1)	19.4(2.3) 26(2.6)	138.41	<0.001 ***	0.7	1	20.8(2.2) 25.5(2.4)	85.25	<0.001 ***	0.6	1	20.1(1.6) 25.8(1.8)
	NHPT pre NHPT pos	244(10.3) 199(10.9)	91.02	<0.001 ***	0.6	1	300(10.3) 300(10.9)	0	1 ns	0	0.05	272(7.3) 249.4(7.7)	275(12) 254(15)	10.43	<0.01 **	0.2	0.9	269(11) 245(14)	14.78	<0.001 ***	0.02	1	272(8) 250(10)
	BBT pre BBT pos	6.7(1.1) 11.9(1.3)	69.58	<0.001 ***	0.6	1	0(1.1) 0.1(1.3)	0.012	0.91 ns	0	0.05	3.3(0.8) 6(0.9)	3.6(1.3) 6.5(1.8)	12.62	<0.01 **	0.2	0.9	3.2(1.2) 5.6(1.7)	9.91	0.003 **	0.15	0.9	3.4(0.9) 6(1.2)
Synergistic Treatment (A + B) COHT + RAT (6 months)	FMUL pre FMUL pos	29.1(10.8) 47.7(9.8)	220.44	<0.001 ***	0.8	1	11.2(2.8) 21.1(8.9)	62.94	<0.001 ***	0.5	1	20.2(11.9) 34.4(16.3)	6.5(0.5) 9.8(1)	25.84	<0.000 ***	0.3	1	4.8(0.5) 7.9(0.9)	27.92	<0.001 ***	0.3	1	5.6(0.4) 8.9(0.7)
	NHPT pre NHPT pos	244(79) 126(115)	135.04	<0.001 ***	0.7	1	300(0) 300(0)	0	0.7 ns	0	0.5	272(62) 213(119)	21.3(6.6) 39(11)	12.53	<0.01 **	0.2	0.9	23.9(6.2) 34(10)	5.03	0.029 *	0.01	0.6	23(4.5) 37(7.6)
	BBT pre BBT pos	6.7(8.4) 22.9(15.4)	87.88	<0.001 ***	0.6	1	0.00(0.00) 0.00(0.00)	0	0.5 ns	0	0.5	3.3(6.8) 11.4(15.8)	2.9(1) 4.9(1.4)	4.77	0.033 *	0.1	0.6	3.7(1) 5.5(1.3)	4.64	0.036 *	0.1	0.6	3.3(0.7) 5.2(0.9)

Descriptive F test values with respective degrees of freedom, effect sizes (η^2^) and statistical power (OP) are shown. BBT: Box and Block Test; COHT: conventional hand-specific occupational therapy; F:ANOVA F test with degrees of freedom; FMUL: Fugl Meyer Upper Limb Scale; M:mean; NHPT: Nine Hole Peg Test; PPP: positive prognostic profile subgroup; PPN: negative prognostic profile subgroup; RAT: robot-assisted therapy; RH: right hemiparesis; LH: left hemiparesis; SD: standard deviation; η^2^: effect size; OP: statistical observed power; *p* value: alpha level of significance; ns: not significant; * *p* < 0.05, ** *p* < 0.01, *** *p* < 0.001.

**Table 3:** ADL functional effects of COHT /RAT treatments at 3 and 6 months by both prognosis (PPP, PPN) and lateralization-cognition groups (RH, LH).

		Hand Hemiparesis at Admission											Lateralization-Cognition										
Intervention	AMPS© Measure Variables	PPP Group *n* = 29 M(SD)	F(1,56)	*p* Value	η^2^	OP	PPN Group *n* = 29 M(SD)	F(1,56)	*p* Value	η^2^	OP	Total *N* = 58 M(SD)	RH Group *n* = 27 M(SD)	F(1,56)	*p* Value	η^2^	OP	LH Group *n* = 31 M(SD)	F(1,56)	*p* Value	η^2^	OP	Total *N* = 58 M(SD)
Experimental Treatment (A) RAT (3 months)	HM-AMPSpre	0.62(0.2)	208.04	<0.001 ***	0.8	1	−1.1(0.2)	61.53	<0.001 ***	0.5	1	−0.24(0.1)	−0.22(0.3)	88.81	<0.001 ***	0.6	1	−0.25(0.2)	90.65	<0.001 ***	0.6	1	−0.24(0.2)
	HM-AMPSpos	1.42(0.2)					−0.67(0.2)					0.38(0.2)	0.41(0.3)					0.34(0.3)					0.38(0.2)
	HP-AMPSpre	−0.12(0.2)	116.38	<0.001 ***	0.7	1	−0.99(0.2)	121.41	<0.001 ***	0.7	1	−0.55(0.1)	−0.65(0.2)	106.56	<0.001 ***	0.7	1	−0.47(0.2)	131.49	<0.001 ***	0.7	1	−0.56(0.1)
	HP-AMPSpos	0.55(0.2)					−0.31(0.2)					0.11(0.1)	0.01(0.2)					−0.21(0.2)					0.11(0.1)
Control Treatment (B) COHT (3 months)	HM-AMPSpre HM-AMPSpos	0.05(0.2) 0.62(0.2)	421.09	<0.001 ***	0.9	1	−1.42(0.2) −1.1(0.2)	132.72	<0.001 ***	0.7	1	−0.68(0.1) −0.24(0.1)	−0.69(0.3) −0.22(0.3)	155.11	<0.001 ***	0.7	1	−0.7(0.2) −0.25(0.2)	147.11	<0.001 ***	0.7	1	−0.68(0.8) −0.24(0.2)
	HP-AMPSpre HP-AMPSpos	−0.7(0.2) −0.1(0.2)	150.7	<0.001 ***	0.7	1	−1.6(0.2) −1(0.2)	127.8	<0.001 ***	0.7	1	−1.2(0.1) −0.6(0.1)	−1.3(0.2) −0.6(0.2)	129.60	<0.001 ***	0.7	1	−1.1(0.2) −0.5(0.2)	146.16	<0.001 ***	0.7	1	−1.2(0.1) −0.6(0.1)
Synergistic Treatment (A + B) COHT + RAT (6 months)	HM-AMPSpre HM-AMPSpos	0.06(1.16) 1.4(1.07)	182.9	<0.001 ***	0.7	1	−1.5(1.2) 0.10(1.06)	172.9	<0.001 ***	0.7	1	−0.70(1.4) 0.76(1.2)	−0.69(0.3) 0.41(0.3)	120.94	<0.001 ***	0.7	1	−0.68(0.2) 0.34(0.28)	119.69	<0.001 ***	0.7	1	−0.68(0.2) 0.38(0.2)
	HP-AMPSpre HP-AMPSpos	−0.75(0.86) 0.54(0.83)	121.6	<0.001 ***	0.8	1	−1.6(1.1) −0.31(0.95)	160.9	<0.001 ***	0.8	1	−1.1(1.1) 0.12(0.98)	−1.3(0.2) 0.01(0.2)	162.91	<0.001 ***	0.7	1	−1.1(0.2) 0.21(0.2)	192.61	<0.001 ***	0.8	1	−1.2(0.1) 0.11(0.1)

Descriptive F test values with respective degrees of freedom, effect sizes (η^2^) and statistical power (OP) are shown. AM: active range of motion; FF: grip flexor strength; EF: release extensor strength; %: percentage of AM; N: Newtons of force; COHT: conventional hand-specific occupational therapy; M: mean; SD: standard deviation; PPP: positive prognostic profile subgroup; PPN: negative prognostic profile subgroup; RAT: robot-assisted therapy; RH: right hemiparesis; LH: left hemiparesis; η^2^: effect size; OP: statistical observed power; F: ANOVA F test with degrees of freedom; ns: not significant; *** *p* < 0.001.

**Table 4:** Parametric correlations between criterion and predictor variables for the Amadeus-A and Amadeus-B regression models.

Criterion Variables	Predictive Variables	N	Mean (SD)	Pearson Coefficient	*p*-Value
Amadeus—A Functional Models					
Amadeus-A1 Predictive Model (AMPS)					
HMAmpsImprov COHT (to 3 months)		58	0.44(0.19)		
	AM_improv_1d	58	3.67(3.53)	0.64	<0.001 ***
	FF_improv_1d	58	2.06(1.77)	0.63	<0.001 ***
	EF_improv_1d	58	0.45(0.52)	0.58	<0.001 ***
Amadeus-A2 Predictive Model (AMPS)					
HMAmpsImprov RAT (to 3 months)		58	0.60(0.33)		
	AM_improv_1d	58	3.67(3.53)	0.46	<0.001 ***
	FF_improv_1d	58	2.06(1.77)	0.49	<0.001 ***
	EF_improv_1d	58	0.45(0.52)	0.59	<0.001 ***
Amadeus-A3 Predictive Model (AMPS)					
HMAmpsImprov COHT + RAT (to 6 months)		58	1.04(0.50)		
	AM_improv_1d	58	3.67(3.53)	0.55	<0.001 ***
	FF_improv_1d	58	2.06(1.77)	0.56	<0.001 ***
	EF_improv_1d	58	0.45(0.52)	0.61	<0.001 ***
AMADEUS-B SENSORIMOTOR MODELS					
Amadeus-B1 Predictive Model (FMUL)					
FMUL_6m COHT + RAT (to 6 months)		58	34.41(16.29)		
	AM_improv_1d	58	3.67(3.53)	0.56	<0.001 ***
	FF_improv_1d	58	2.06(1.77)	0.64	<0.001 ***
	EF_improv_1d	58	0.45(0.52)	0.77	<0.001 ***
Amadeus-B2 Predictive Model (BBT)					
BBT_6m COHT + RAT (to 6 months)		58	11.45(15.81)		
	AM_improv_1d	58	3.67(3.53)	0.44	<0.001 ***
	FF_improv_1d	58	2.06(1.77)	0.60	<0.001 ***
	EF_improv_1d	58	0.45(0.52)	0.78	<0.001 ***
Amadeus-B3 Predictive Model (NHPT)					
NHPT_6m COHT + RAT (to 6 months)		58	212.84(119.37)		
	AM_improv_1d	58	3.67(3.53)	−0.48	<0.001 ***
	FF_improv_1d	58	2.06(1.77)	−0.63	<0.001 ***
	EF_improv_1d	58	0.45(0.52)	−0.78	<0.001 ***

AM: active range of motion; AMPS: assessment of motor and process skills measure; COHT: conventional occupational hand treatment; EF: extension force hand grasp; FF: flexion force hand grasp; N: sample; SD: standard deviation; RAT: robotic assisted treatment; 1d: first assessment day; *** *p* < 0.001.

**Table 5:** Backward stepwise multiple linear regression analysis for the predictive models Amadeus-A and Amadeus-B at3 and 6 months post-intervention based on the evaluation of the effect of the first pre-intervention session.

		Regression Coefficient (B)	Standarized Coefficient (β)			Overall Model			Analysis of Residuals	
Predictive Models				*p*-Value	VIF	R	Adjusted R^2^	F	Durbin-Watson	D
Amadeus-A Functional models										
Amadeus-A1 Predictive Model (AMPS)										
VD: HMAmps COHT(to 3months)				<0.001 *		0.72	0.51	29.03	1.7	0.49
	VP: AMimprov1d FFimprov1d	0.23 0.43	0.41 0.39	0.001 * 0.001 *	1.50 1.50					
	VE: EFimprov1d		0.22	0.071	1.68					
Amadeus-A2 Predictive Model (AMPS)										
VD: HMAmps RAT(to 3months)				<0.001 *		0.59	0.34	30.32	1.7	0.19
	VP:									
	EFimprov1d	0.37	0.59	<0.001 *	1.00					
	VE:									
	AMimprov1d FFimprov1d		0.20 0.22	0.12 0.10	1.42 1.51					
Amadeus-A3 Predictive Model (AMPS)										
VD: HMAmps COHT + RAT(to 6months)				<0.001 *		0.67	0.43	22.23	1.8	0.38
	VP:									
	EFimprov1d	0.43	0.45	<0.001 *	1.42					
	AMimprov1d	0.04	0.31	0.012 *	1.42					
	VE:									
	FFimprov1d		0.22	0.09	1.77					
AMADEUS-B Sensorimotor Models										
Amadeus-B1 Predictive Model (FMUL)										
VD: FMUL_6m (to 6 months)				<0.001 *		0.80	0.64	51.09	1.4	0.12
	VP:									
	FFimprov1d	2.7	0.29	0.004 *	1.51					
	EFimprov1d	18.6	0.60	<0.001 *	1.51					
	VE:									
	AMimprov1d		0.10	0.32	1.66					
Amadeus-B2 Predictive Model (BBT)										
VD:BBT_6m (to 6 months)				<0.001 *		0.80	0.62	48.11	1.5	0.22
	VP:									
	FFimprov1d	2.02	0.23	0.028 *	1.51					
	EFimprov1d	19.48	0.65	<0.001 *	1.51					
	VE:									
	AMimprov1d		−0.07	0.51	1.67					
Amadeus-B3 Predictive Model (NHPT)										
VD:NHPT_6m (to 6 months)				<0.001 *		0.81	0.65	53.05	1.6	0.25
	VP:									
	FFimprov1d	−18.36	−0.27	0.007 *	1.51					
	EFimprov1d	−141.86	−0.62	<0.001 *	1.51					
	VE:									
	AMimprov1d		0.26	0.78	1.67					

AM: active range of motion(%) with robot; AMPS: assessment of motor and process skills measure; B: regression coefficient; β: standardized regression coefficient; BBT: box and block test; CT: conventional OT treatment; D: Highest value of Cook´s Distances; EF: extension force of hand graspin Newton’s (N) with the robot; FF: flexion force of hand grasp in Newton’s (N) with the robot; FMUL: Fugl-Meyer upper limb scale; HM: motor skills; NHPT: nine hole peg test; RAT: robot assisted OT treatment; VD: dependent variable; VE: excluded variable; VIF: variance inflation factor; VP: predictor variable; 1d: first assessment day; 3m: 3 months; 6m: 6 months. Table shows *p*-values of ANOVA test: * *p* < 0.05.

## Data Availability

Archived offline at the Beata María Ana´s Hospital, under the custody of the principal investigator.

## Abbreviations

ABI	Acquired Brain Injury
ADLs	Activities of Daily Living
AHR	Amadeo Hand Robot
AM	Active range of Motion
AMPS	Assessment of Motor and Process Skills
BBT	Box and Block Test
COHT	Conventional Occupational Hand Therapy
EEG	Electroencephalography
EF	Extension Force
FMUL	Fugl Meyer Upper Limb
FF	Flexion Force
HM-AMPS	Motor Skills-AMPS
HP-AMPS	Process Skills-AMPS
NHPT	Nine Hole Peg Test
OT	Occupational Therapy
PT	Physiotherapy
RAT	Robot Assisted Therapy
sEMG	Surface Electromyography
UL	Upper limb
VR	Virtual Reality
